# Promoting Self-Management and Independent Living of Older Individuals with Chronic Diseases through Technology: A Study of Self-Reported Needs, Priorities, and Preferences

**DOI:** 10.3390/medicina59081493

**Published:** 2023-08-19

**Authors:** Argyroula Kalaitzaki, Michael Rovithis, Alexios Dimitropoulos, Sofia Koukouli, Manolis Linardakis, Elli Katsiri, Nikos Rikos, George Vasilopoulos, George Tsolas, Aikaterini Papachristou, Anastasia Dimitrantzou, Dimitrios Katsiris, Areti Stavropoulou

**Affiliations:** 1 Department of Social Work, School of Health Sciences, Hellenic Mediterranean University, Gianni Kornarou, Estavromenos 1, 71410 Heraklion, Greecekoukouli@hmu.gr (S.K.); 2 Laboratory of Interdisciplinary Approaches for the Enhancement of Quality of Life (QoLab), 71410 Heraklion, Greece; rikosn@hmu.gr (N.R.); astavropoulou@uniwa.gr (A.S.); 3 Department of Business Administration and Tourism, School of Management and Economics Sciences, Hellenic Mediterranean University, Gianni Kornarou, Estavromenos 1, 71410 Heraklion, Greece; 4 TECHAPPS HEALTHIER IKΕ, 56 Ippokratous Str., 10680 Athens, Greece; alexios.dimitropoulos@gmail.com (A.D.); anastasiad25@gmail.com (A.D.); 5 Department of Social Medicine, Faculty of Medicine, University of Crete, Andrea Kalokerinou 13, Giofirakia, 71500 Heraklion, Greece; linman@med.uoc.gr; 6 Innosense ΙΚΕ, 18 Esperidon Str., 13674 Athens, Greece; ekatsiri@gmail.com (E.K.); dkatsiris@gmail.com (D.K.); 7 Department of Nursing, Faculty of Health Sciences, Hellenic Mediterranean University, Gianni Kornarou, Estavromenos 1, 71410 Heraklion, Greece; 8 Department of Nursing, Faculty of Health and Care Sciences, University of West Attica, Ag. Spyridonos Str., 12243 Athens, Greece; gvasilop@uniwa.gr; 9 Institute of Nursing Research and Health Policy, 73 Aristotelous Str., 10434 Athens, Greece; georgetsolas@gmail.com (G.T.); kpapachri@hotmail.com (A.P.); 10 Faculty of Health, Science, Social Care and Education, Kingston University, KT2 7LB London, UK

**Keywords:** chronic non-communicable diseases, disease management, digital health technologies, technology-based applications, mHealth, mobile apps

## Abstract

*Background and Objectives*: Older patients’ needs are rarely examined beforehand, and thus, although technology-based tools can enhance self-management, acceptability rates are still low. This study aimed to examine and compare self-reported needs, priorities, and preferences of older patients with heart failure (HF), diabetes mellitus type II (DM2), and chronic obstructive pulmonary disease (COPD) toward technology use to enhance self-management. *Materials and Methods*: A convenience sample of 473 participants over 60 s (60.5% females), diagnosed with HF (*n*= 156), DM2 *(n* = 164), or COPD *(n* = 153) was recruited. They were administered a questionnaire about the usefulness of technology in general and in specific areas of disease management. *Results*: Most participants (84.7%) admitted that technology is needed for better disease management. This was equally recognized across the three groups both for the overall and specific areas of disease management (in order of priority: “Information”, “Communication with Physicians and Caregivers”, and “Quality of Life and Wellbeing”). Sociodemographic differences were found. Cell phones and PCs were the devices of preference. The four common features prioritized by all three groups were related to ‘information about disease management’ (i.e., monitoring symptoms, reminders for medication intake, management and prevention of complications), whereas the fifth one was related to ‘communication with physicians and caregivers (i.e., in case of abnormal or critical signs). The top disease-specific feature was also monitoring systems (of respiratory rate or blood sugar or blood pressure, and oxygen), whereas other disease-specific features followed (i.e., maintaining normal weight for HF patients, adjusting insulin dose for DM2 patients, and training on breathing exercises for COPD patients). *Conclusions*: Older individuals in these three groups seem receptive to technology in disease management. mHealth tools, incorporating both common and disease-specific features and addressing different chronic patients, and also being personalized at the same time, could be cost-saving and useful adjuncts in routine clinical care to improve self-management.

## 1. Introduction

Health prevention and promotion of the older population (age above 60 years old) are the most important public health challenges that Western societies will have to face in the coming years. Alongside the growing life expectancy, the incidence and prevalence of chronic non-communicable diseases (CNCDs; e.g., cardiovascular diseases, chronic respiratory diseases, and diabetes mellitus) are reaching an epidemic level, with 71% of deaths each year [1]. The increasing numbers of CNCDs compromise people’s productivity and quality of life, and result in steadily increasing healthcare utilization and, therefore, excessive national healthcare expenditures [2].

Self-management (e.g., disease monitoring, medication intake, decision making, lifestyle modifications) [3] enhances patients’ autonomy and sense of control over the disease, and it is considered one of the most important factors in ensuring health prevention and promotion. It is associated with adherence to therapy, reduction in hospitalizations, decreased healthcare costs, improved health outcomes, and quality of life [1,4]. However, actively engaging chronic patients in self-management has been difficult [5]. Digital health technologies (DHT), such as web-based solutions, eHealth, and mHealth (i.e., mobile apps), are promising, patient-centered, and cost-effective tools that offer the possibility of self-management of CNCDs (e.g., improve disease monitoring, increase adherence, promote healthy lifestyles, and improve health-related behaviors) [6]. DHT has been associated with improved quality, accessibility, cost, and efficiency of healthcare, such as reductions in mortality rates, hospitalizations, and readmissions [7], along with substantial improvements in independent living, adherence, and quality of life [8].

Although the unprecedented speed of proliferation and availability of DHT has made them abound nowadays, and relevant studies have suggested DHT to be a useful facilitator in the prevention or management of CNCDs, particularly in older patients [9], acceptance and adoption rates remain relatively low among the older people [10]. The studies on the effectiveness of DHT in self-management have been limited [11] and, though promising [12], have not yet provided convincing evidence, and results are rather mixed and inconsistent [13,14,15]. Old age is intertwined with features such as low health literacy and limited smartphone experience [16,17,18], and older people typically have low adherence, low eHealth use [13,19], and low commitment levels. Though reluctantly curious, they have also been shown to be ambivalent towards e-health [20].

Understanding and accounting for older people’s needs and preferences in the development of an mHealth tool prior to its design could potentially enhance DHT benefits by increasing acceptance, commitment, and clinical outcomes [21,22]. However, research on older patients’ needs has been lacking, and it typically focuses either on specific diseases or on other age groups [23]. Studies mainly examine features and effectiveness (feasibility and usability, and health outcomes) of mHealth tools [24,25,26] once they have been designed and used [15] as well as barriers and facilitators in using apps (intention, acceptance), such as the characteristics of a device (e.g., user-friendly interface, intriguing features) [10,27], and very few have examined older patients’ attitudes, beliefs, experiences, and expectations towards e-health services as factors influencing commitment to such tools. [20]. Older patients’ self-reported needs are rarely addressed or prioritized in the design or implementation phases of DHT, and few applications have been evidence-based, which means that analysis studies are needed [28].

Therefore, the purpose of this study was to examine the self-reported needs, priorities, and preferences of older individuals (≥60 years) suffering from chronic diseases toward technology use in order to enhance autonomy in disease management and explore potential differences between three chronic diseases (i.e., heart failure, diabetes mellitus, and chronic obstructive pulmonary disease).

## 2. Materials and Methods

This study was conducted within the framework of the project entitled Empowered: An Integrated, Intelligent Assistant to Support Independent Living for older Patients, funded by the action RESEARCH-CREATE-INNOVATE Operational Programme Competitiveness, Entrepreneurship, and Innovation (ESPA 2014–2020) (EPAnEK) (Ref. No 5070922/29-9-2020).

### 2.1. Study Design and Participants

This is a cross-sectional study, conducted from May to July 2022. Eligibility criteria were: (1) older patient primarily diagnosed with one of the following diseases: heart failure (HF), type II diabetes mellitus (DM2), or chronic obstructive pulmonary disease (COPD); (2) aged 60 years and above; and (3) without any cognitive impairment. A sample of 473 people diagnosed with either heart failure (N = 156, 33.0%) diabetes mellitus type II (N = 164, 34.7%) or chronic obstructive pulmonary disease (N = 153, 32.3%) participated in the study. They were mostly females (60.5%), 71–80 years old (33.4%), single or divorced (52.9%), unemployed (59.4%), and had elementary education (35.1%). The detailed socio-demographics of the three groups can be found in Table 1.

### 2.2. Instrument

A questionnaire was developed by the researchers, which included two sections that were common among the three groups of patients. There were a total of 10 socio-demographic questions and 21 questions allocated to the three subgroups about patients’ views on the extent that the use of technology could be useful in various aspects of disease management: quality of life/well-being (8 questions, such as dealing with stress and depression, and reminders for health-enhancing behaviors, such as exercise, diet, medical appointments, positive reinforcement for achieving disease improvement goals); information and disease management (7 questions, such as monitoring of current health status, reminders to take medication, and prevention and management of disease-related complications); and communication with physicians and caregivers (6 questions, such as warning and caregiver notification when necessary or immediate medical attention being required). Cronbach’s alpha was also calculated for each subgroup of questions: for Quality of Life/Well-being α = 0.746 (8 items), for Information and Disease Management α = 0.791 (7 items), and for Communication with Physicians and Caregivers α = 0.772 (6 items) (Table 2). Additionally, a third section was selectively administered to the patients, depending on their disease, to record their views on whether technology use can help various aspects of the disease-specific management: heart failure patients (4 questions; e.g., maintain a normal weight, monitor respiratory rate), diabetes mellitus patients (7 questions; e.g., monitoring of vision, blood sugar, blood pressure, cholesterol levels, adjustment of insulin dose), and chronic obstructive pulmonary disease patients (10 questions; e.g., increase fluid intake, coping with shortness of breath, use of monitoring devices for blood pressure, oxygen, etc.). A five-point Likert-type scale, ranging from ‘not at all’ to ‘very much’, was used to rate responses.

### 2.3. Procedure

A convenience sampling method was used for data collection since researchers had access to potential participants. Participants were recruited from health facilities (4 nursing homes and 3 medical centers) and the questionnaire was administered through interviews or individually using a paper-and-pencil form of the questionnaire. The mean time to complete the questionnaires was approximately 15 min. There was no compensation for participating in the study. Ethics and confidentiality were assured throughout the study process. The study was conducted in accordance with the Declaration of Helsinki and approved by the Hellenic Mediterranean University Ref. No. of approval 74/θ.21/18.11/2020).

### 2.4. Statistical Analysis

Statistical analysis was conducted using IBM SPSS Statistics for Windows, v.26.0, IBM Corp: Armonk, NY, USA). Self-management of the disease was assessed with three questions: the perceived ability of self-management, the need for support on disease management, and the need for technology to improve disease management. The usefulness of technology was assessed in three areas of disease management: Quality of Life/Well-being (8 items), Information and Disease Management (78 items), and Communication with Physicians and Caregivers (6 items). For the comparison of frequency distributions, the 95% confidence intervals (95% CI) were calculated, respectively, while the differences in response distributions in the aspects of disease management (i.e., Quality of Life and Well-Being, Information and Management of the Disease, and Communication with Physicians and Caregivers) were estimated through the Friedman method. Their validity and reliability were determined through independent cross-questioning. The shape of the distributions of the scores of the three aspects of disease management was checked through the Blom method (QQ plot), and due to slight asymmetry, their univariate correlation followed with the Pearson parametric method both among themselves and with the characteristics of the participants. This was followed by an analysis of variance test between the three aspects of disease management and Student t regarding the categories of the characteristics. An acceptable level of significance was set at 0.05.

## 3. Results

### 3.1. Self- or Supported Management of Disease

In Table 1 can be seen that, although most of the participants (nearly 90%) reported that they moderately (42.9%) or very much (46.3%) could manage their disease satisfactorily, the majority (nearly 65%) also reported that they need moderately (30.2%) or very much (34.5%) support to this. Nearly 85% believed that the use of technology (e.g., a smartphone application) can better help disease management, either moderately (30.2%) or very much (54.5%).

#### 3.1.1. Usefulness of Technology in Disease Management

The participants reported a significantly higher need for technology use in the area of Information and Disease Management (*M* = 3.57) and Communication with Physicians and Caregivers (*M* = 3.51) compared to Quality of Life and Wellbeing (*M* = 3.38) (*p* < 0.001). The detailed responses, hierarchically, in each of the three areas of disease management can be found in Table 2.

#### 3.1.2. Usefulness of Technology and Sociodemographic Characteristics

Those with higher (i.e., postgraduate) education (3.90 ± 0.49), those employed (3.57 ± 0.51), and those with a very good financial situation (3.42 ± 0.45) reported significantly higher scores in the usefulness of technology in Information and Disease Management, Communication with Physicians and Caregivers, and Quality of Life and Well-being, respectively, compared to those with lower education (Elementary, 3.47 ± 0.53; *p* < 0.05), not being employed (3.46 ± 0.54; *p* < 0.05), and with a bad financial situation (3.25 ± 0.50; *p* < 0.05) (results not shown in table/figure).

### 3.2. Usefulness of Technology in Disease Management across the Three Groups of Patients

High mean scores on the usefulness of technology in disease management (overall and its three aspects) were found for the three groups of patients (scores ranging from 3.4 to 3.6). However, no significant differences were found between the three groups of patients in either the overall disease management score or in its three aspects (results not shown in table/figure).

#### 3.2.1. Device Preference for Disease Management

The rates of patients reporting the device of their preference for helping them in disease management can be seen in Table 3. Cell phones and PCs outweighed the rest of the devices. DM2 and COPD patients prioritized the cell phone over the rest of the devices and, secondly, the PC, whereas the rates for HF patients were vice versa.

#### 3.2.2. Preference of Disease-Specific Features for Disease Management

In Table 4, it can be seen that the HF patients reported the highest mean score on ‘record/monitor of respiratory rate and single-lead electrocardiogram’ (3.60 ± 1.09) and the lowest on ‘appropriate exercise program’ (3.46 ± 0.87) (*p* > 0.05). The DM2 patients reported the highest mean scores on‘ measuring/monitoring blood sugar’ (3.84 ± 1.00) and the lowest on‘ monitoring/control of weight’ (3.46 ± 1.13) (*p* = 0.004). The COPD patients reported the highest mean scores on‘ ue of monitoring devices/equipment’ (3.73 ± 0.97) and the lowest on‘ increased fluid intake’ (3.39 ± 1.01) (*p* > 0.05).

## 4. Discussion

This study aimed at exploring the needs, priorities, and preferences of three groups of older patients suffering from chronic diseases regarding the usefulness of technology in enhancing disease management in three areas (quality of life/well-being, information, and communication with physicians and caregivers).

Although most of the participants reported that they could manage their disease satisfactorily in general, the majority also reported that they still needed support for this. Consistent with others [29], these findings show that older patients have unmet needs in several domains of disease management. These unmet needs could comprise the targets of relevant interventions and application development. Pertinent to this is the equally reported belief in the usefulness of technology (e.g., a smartphone application) for better disease management by the three sampling groups. This is a noteworthy and reassuring finding which suggests that old people in these three groups are receptive to the use of technology in the management of their disease, although contradictory evidence from the relevant literature demonstrates that the use of technology-based applications among users of any age in healthcare is very low [30], and old people have even lower rates [9] due to limited support, access, skills, and knowledge in the use of m-Health technologies.

The examination of demographics showed that those who were well educated prioritized the need for technology use in the area of information, and those employed prioritized the need for technology use in the area of communication with physicians and caregivers as well as those with very good financial situations (in the area of quality of life and well-being). The findings were in line with others that have shown that occupational status, high income, and education are associated with high eHealth use [13,19]. Well-educated people seem to value knowledge and information to facilitate disease management. People with a high income seem to have the resources that allow them to ensure information and access to healthcare, and, thus, well-being is a priority. Reasons for preference of the area of communication with physicians and caregivers by those employed are less clear; however, it could be due to time constraints.

### 4.1. Common and Specific Features

Respondents in all three groups of patients equally identified that technology could be useful in overall disease management and in its three aspects. Rather quite plausibly, “Quality of Life and Well-being” followed the other two first-choice areas of disease management. Participants prioritized technology use in providing information and secondly communication with physicians and caregivers.

The areas of disease management that participants identified as struggling most and needing help from technology were both a group of common and a group of disease-specific features. As far as the common features are concerned, it is noteworthy that four features related to information for disease management received the highest scores and outweighed the others, such as the use of monitoring devices/equipment (e.g., blood pressure, oxygen, glucose, etc.), reminders for medication intake, management of disease-related complications (e.g., diabetic foot care, infections for COPD, etc.), and prevention of disease-related complications (e.g., foot control, infections for COPD, etc.). Not surprisingly, the need for technology-based options for the three CNCDs prioritized monitoring systems [14,31,32]. Prevention and management of complications through mHealth, such as diabetic foot, have received a lot of attention [33], and medication adherence is one of the top priorities in health apps [10,34,35]). The fifth feature came from the communication with physicians and caregivers’ area (i.e., notifying the caregiver when there are abnormal or critical signs). To the authors’ knowledge, the involvement of the caregiver has been predominately examined in educational interventions of increasing their familiarity with technology [35], whereas Slevin found that COPD patients believed that data derived from DHT could potentially facilitate their interaction with physicians [5]. Fears and insecurity about an unexpected health problem or complication could also explain this finding.

Although HF, DM2, and COPD are related to some extent to behavioral risk factors resulting from unhealthy lifestyle habits such as smoking, unhealthy diet, and physical inactivity/sedentary lifestyle, which can be modified and be benefited from the use of technology [13,35], health-promoting behaviors (e.g., adoption of proper nutrition and stop smoking) were least prioritized by the patients. This finding indicates that there is a need to address potential underlying deficiencies in older people’s willingness and ability to understand, prioritize, and promote healthy behaviors. Health literacy skills [16], coping mechanisms [36], self-efficacy, patient activation, and motivation [37] have also been shown as important to adherence to disease-related self-care activities by older people with chronic diseases living at home. The study of the pathway and the mediating variables through which self-management is achieved seems to be important for future research.

The device of preference is cell phones for DM2 and COPD patients, and PC for HF patients. This result may be attributable to the distinct interface of the health applications pertaining to HF patients, as health data monitoring is more complicated, and the patient can manage them more effectively on a computer screen rather than on a mobile phone.

In terms of the disease-specific features, the findings were as anticipated [14,31,32,35]: Specific monitoring systems were the first choice by all three groups of patients (recording/monitoring respiratory rate and single-lead electrocardiogram for HF patients [31,35], measuring/monitoring blood sugar for DM2 [32], and monitoring devices/equipment for blood pressure, oxygen, glucose, etc. for COPD patients [14]), whereas other disease-specific features followed: maintaining normal weight for HF patients, adjusting insulin dose according to glucose levels for DM2 patients, and training on breathing exercises for COPD patients. It can be assumed that older individuals have limited access to healthcare resources and, thus, their need for remote monitoring is increased. For this, a vast number of monitoring devices have been developed for these diseases [14,22,28,38].

### 4.2. Limitations

Specific limitations can be mentioned in relation to this study. The convenient sample limits the generalizability of the findings, resulting in a low external validity in the present study. Since this sampling technique imposes limits on generalizability, the results of this study should be viewed under this limitation. Although there was a small percentage of patients with comorbidity (ranging from 2.1% to 5.5%; see Table 2), these were not excluded from the sample, and this could be a serious limitation. Even though the patients were instructed to specifically respond to the questionnaire in terms of their major disease, comorbidity could be a confounding factor that we need to recognize. Additionally, we do not know whether and to what extent participants had access to healthcare, help from informal caregivers, prior or current experience, and skills of technology use or what their perceptions and attitudes were towards health apps or technology in general, and all of these may have impacted participants’ attitudes about the importance and usefulness of technology in disease management.

Despite the limitations, the study demonstrated useful insights. One strong point of this study was that it explored patients’ needs before developing any mHealth tool, which could guide individualized and tailored solutions. A second one is that the study examined multiple chronic diseases, rather than one, which adds to the understanding of the patients’ common and unique needs [25]. Given the satisfactory number of participants in each group of patients, comparisons between the groups were also allowed. The findings of this study may inform the design of apps to simultaneously address multiple chronic patients and be individualized according to their needs and preferences [39].

## 5. Conclusions

The findings of this study showed that older patients with chronic diseases (DM2, COPD, HF) perceive the need for technology-based tools, such as mobile apps, to enhance self-management, improve their independence, and help them achieve optimal health outcomes. Findings also suggest that both common and disease-specific features could be incorporated into mobile apps to improve self-reported management deficits. Mobile apps can be an easy and accessible way to track symptoms and medication adherence and connect with healthcare providers and caregivers when needed.

Technology and mHealth interventions in general can serve as effective alternatives that increase self-management, and if they are accepted by the users, they may be useful adjuncts in routine clinical care. They could be incorporated into an integrated management plan by physicians, nurses, and allied health practitioners to increase self-management and enrich care both within and outside the clinical setting. Technologically-based self-management solutions can be cost-effective and alleviate significant burdens on professionals, caregivers, and healthcare services in the long run. However, in order to be effective, apps should be based on evidence [10] and align with patients’ personalized self-management goals and plans [25]. These features may increase patients’ engagement in self-management [21,22]. Further research is recommended using a random sampling technique. Examining possible correlations among perceived self-management needs and how technology may support older patients with chronic diseases will expand our knowledge in this field.

## Figures and Tables

**Table 1:** Sociodemographic characteristics of the three groups.

		Total Sample (N = 473)		Heart Failure (N = 156)		Diabetes Mellitus Type II (N = 164)		Chronic Obstructive Pulmonary Disease (N = 153)	
		n	%	n	%	n	%	n	%
Gender	Male/Female	187/286	39.5/60.5	63/93	40.4/59.6	67/97	40.9/59.1	57/96	37.3/62.7
Age (years)	60–70	147	31.1	56	35.9	51	31.1	40	26.1
	71–80	158	33.4	50	32.1	57	34.8	51	33.3
	81–90	133	28.1	43	27.5	44	26.8	46	30.1
	>90	35	7.4	7	4.5	12	7.3	16	10.5
Family status	Married, Cohabited	223	47.1	83	53.2	74	45.1	66	43.1
	Single, Divorced	250	52.9	73	46.8	90	54.9	87	56.9
Education	Up to Elementary	166	35.1	54	34.6	56	34.1	56	36.6
	Junior high school	120	25.4	31	19.9	43	26.2	46	30.1
	High school	83	17.5	31	19.9	31	18.9	21	13.7
	University/College	82	17.3	32	20.5	28	17.1	22	14.4
	Postgraduate studies	22	4.7	8	5.1	6	3.7	8	5.2
Financial situation	Bad	40	8.5	9	5.8	18	11.0	13	8.5
	Moderate	224	47.4	74	47.4	68	41.5	82	53.6
	Good/Very Good	209	44.2	73	46.8	78	47.6	58	37.9
Employment (yes)		192	40.6	66	42.3	67	40.9	59	38.6
Disease	Heart failure	156	33.0	144	30.4				
	Diabetes mellitus Type II	164	34.7	22	4.7	145	30.7		
	Chronic obstructive pulmonary disease	153	32.3	10	2.1	26	5.5	126	26.6
Perceived self-ability/Self-management	Not at all	51	10.8	18	11.6	20	12.2	13	8.5
	Moderately	203	42.9	62	39.7	67	40.8	74	48.4
	Very much	219	46.3	76	48.7	77	47.0	66	43.1
Need for support	Not at all	167	35.3	50	32.1	58	35.4	59	38.6
	Moderately	143	30.2	49	31.4	51	31.1	43	28.1
	Very much	163	34.5	57	36.5	55	33.5	51	33.3
Need for technology	Not at all	74	15.6	22	14.1	23	14.0	29	19.0
	Moderately	142	30.0	51	32.7	55	33.5	36	23.5
	Very much	257	54.4	83	53.2	86	52.5	88	57.5

**Table 2:** Hierarchical distribution of responses to the usefulness of technology in the three areas of self-management (i.e., quality of life/well-being, information and disease management, and communication with physicians and caregivers).

	M	SD
Quality of Life/Well-being (Cronbach Alpha = 0.746)	3.38	0.46
1. Reminders for health-enhancing behaviors (e.g., exercise, diet, medical appointments, etc.)	3.54	1.02
2. Quality of life improvement	3.53	0.95
3. Positive reinforcement for achieving disease improvement goals	3.53	1.04
4. Record of current health status	3.49	0.94
5. Maintaining optimism and hope	3.42	1.03
6. Quality of sleep improvement	3.30	0.92
7. Dealing with stress	3.05	1.15
8. Dealing with depression	2.99	1.08
Information and Disease Management (Cronbach Alpha = 0.791)	3.57	0.50
1. Use of monitoring devices/equipment (e.g., blood pressure, oxygen, glucose, etc.)	3.74	1.00
2. Reminder on medication intake according to medical instructions	3.66	0.94
3. Management of disease-related complications (e.g., diabetic foot care, infections for COPD, etc.)	3.66	1.09
4. Prevention of disease-related complications (e.g., foot control, infections for COPD, etc.)	3.64	1.02
5. Increasing knowledge about the disease	3.57	1.08
6. Education—Adoption of proper nutrition	3.51	1.07
7. Stop smoking	3.02	1.33
Communication with Physicians and Caregivers(Cronbach Alpha = 0.772)	3.51	0.54
1. Notifying the caregiver (family or doctor) when there are abnormal or critical signs	3.60	1.01
2. Warning when immediate medical assistance is required	3.51	1.04
3. Direct contact with the health care system when required	3.50	1.07
4. Increase in perceived support (e.g., an app helps you manage your illness)	3.49	0.99
5. Improving communication with health professionals	3.48	1.01
6. Scheduling visits to the doctor	3.46	1.01

**Table 3:** Device preference for disease self-management.

	HF (N = 156)	DM2 (N = 164)	COPD (N = 153)
Cell phone	26.3	27.4	28.8
PC	27.6	23.2	20.9
Tablet	23.1	22.0	17.6
Smartwatch	12.8	13.4	17.6
Other	10.3	14.0	15.0

Note. Numbers are percentages. HF = Heart failure patients; DM2 = Diabetes mellitus patients; COPD = Chronic obstructive pulmonary disease patients.

**Table 4:** Hierarchical distribution of responses (means and standard deviations) for the preference of disease-specific features for disease management.

	M (SD)
Heart failure patients (HF)	3.53 (0.60)
Record/monitor respiratory rate and single-lead electrocardiogram	3.60 (1.09)
2. Maintaining a normal weight	3.54 (1.03)
3. Appropriate exercise program	3.46 (0.87)
Diabetes mellitus Type II patients (DM2)	3.60 (0.54)
Measuring/monitoring blood sugar	3.84 (1.00)
2. Adjusting insulin dose according to glucose levels	3.73 (1.02)
3. Measuring/monitoring cholesterol	3.59 (1.06)
4. Eye check	3.54 (1.08)
5. Monitoring blood pressure	3.46 (1.13)
6. Monitoring/control of weight	3.46 (0.85)
Chronic obstructive pulmonary disease patients (COPD)	3.53 (0.45)
Use of monitoring devices/equipment (e.g., for blood pressure, oxygen, glucose, etc.)	3.73 (0.97)
2. Training on breathing exercises	3.63 (1.03)
3. Knowledge of the use of an oxygen supply device	3.60 (1.03)
4. Appropriate exercise program	3.56 (0.94)
5. Treating shortness of breath	3.50 (1.06)
6. Weight maintenance	3.50 (1.03)
7. Maintaining optimism and hope	3.44 (1.03)
8. Dealing with the fear of possible shortness of breath	3.42 (1.24)
9. Increased fluid intake	3.39 (1.01)

## Data Availability

Data are available upon request to the corresponding authors.
